# Personal factors understood through the Ecological-Enactive Model of Disability and implications for rehabilitation research

**DOI:** 10.3389/fresc.2022.954061

**Published:** 2022-08-12

**Authors:** Sarah M. Schwab, Caroline Spencer, Nicole S. Carver, Valéria Andrade, Sarah Dugan, Kelly Greve, Paula L. Silva

**Affiliations:** ^1^ Department of Psychology, Center for Cognition, Action & Perception, University of Cincinnati, Cincinnati, OH, United States; ^2^ Department of Speech, Language, and Hearing Sciences, Boston University, Boston, MA, United States; ^3^ Department of Rehabilitation, Exercise, and Nutrition Sciences, University of Cincinnati, Cincinnati, OH, United States; ^4^ Division of Occupational Therapy and Physical Therapy, Cincinnati Children’s Hospital Medical Center, Cincinnati, OH, United States

**Keywords:** rehabilitation, disability, ICF, personal factors, environmental factors, Friedreich's Ataxia

## Abstract

The International Classification of Functioning, Disability and Health (ICF) recognizes that disability arises from the interaction between an individual with a medical condition and the context in which they are embedded. Context in the ICF is comprised of environmental and personal factors. Personal factors, the background life and lifestyle of an individual, are poorly understood in rehabilitation. There is limited knowledge about how personal and environmental factors interact to shape the contextual conditions critical for explaining functioning and disability. In this paper, we explore how a newly proposed model of disability, the Ecological-Enactive Model of Disability, can enhance understanding of personal factors across multiple rehabilitation disciplines. We draw from a review of evidence and phenomenological interviews of individuals with Friedreich's Ataxia. We consider the practical impact of this understanding on disability and rehabilitation research and pathways for the future focusing on representative design.

## Introduction

Models of disability are used across rehabilitation disciplines to promote understanding of what it means to be disabled and identify causes of disability (1). The medical model of disability is predicated on a reductionist, biomechanical understanding of the body (2). Disability in this view is considered the consequence of a pathological medical condition and something to be “fixed” or “normalized” by a rehabilitation professional (3–6). This model places disability *within* the individual, disconnected from the social world (2, 7). On the other hand, the social model of disability seeks to overcome the limitations of the medical model by exclusively emphasizing the role of the social world (8). In this view, disability is understood as an artificial social classification caused by factors *outside* the individual (7). The social model of disability, however, fails to account for how an individual's lived experience of the world is shaped by bodily impairment (8–10). Thus, neither the medical nor the social model concurrently address the interaction between internal and external factors that shape disability.

Rather than reducing the complexity of disability to either an individual's medical condition or their experience in the social world, biopsychosocial frameworks of disability, such as the International Classification of Functioning, Disability and Health (ICF) (11), merge and intertwine the two viewpoints by considering an individual's functioning and disability as a synthesis of biological, individual, and social aspects of health. Human functioning is described at three levels in the ICF: The level of the body (body structures and functions), the level of the whole person in action (activity), and the level of the whole person exercising social roles (participation) (11). Dysfunction at any of these levels is a product of the interaction between an individual with a medical condition and the context in which they are embedded. Accordingly, two individuals with similar medical conditions may exhibit very different patterns of function/dysfunction at the various ICF levels depending on contextual barriers or support they encounter. Thus, a tripartite linear causal chain logic from structural impairment to performance limitations to subsequent disability cannot be expected. Rather, disability is an experience that emerges from the dynamic interaction between function and dysfunction at these various levels, which are shaped by the time-varying context in which the individual is embedded.

Context is defined within the ICF as environmental and personal factors. Environmental factors are considered external to the individual and involve the physical, social, and attitudinal environment in which people live (11). Environmental factors in the ICF are highly structured and provide a comprehensive description of all possible features constituting an environment (12). In contrast, personal factors are less well-defined (i.e., no formal classification scheme) due in part to large social and cultural variance (11). Personal factors include internal factors about a person's life and lifestyle, such as age, gender identity, race, ethnicity, education, religion, socioeconomic status, and occupation, that influence their disability experience (11). Past and current life events, habits, upbringing, and other health conditions are also included within the scope of personal factors (11). Personal factors provide a background useful for understanding how individuals think, understand, and cope with their situation.

Research on the role of contextual factors often emphasizes the environmental factors component of context (12–16), rather than the personal factors component. However, some disability scholars have argued that personal factors are the greatest contributors to chronic illness and disability (17–19), as they can alter an individual's commitment to participation in rehabilitation and explicate an individual's hopes and desires that shape their experience (20). The recognition of the important role that personal factors play on a person's disability experience has been central to ongoing discussions about reconceptualizing the ICF (21, 22).

Despite the substantial impact that personal factors have on the lived experience of disability, there is wide debate regarding the identification of personal factors in rehabilitation literature (20, 23–25), and many factors identified as personal factors can actually be linked to other ICF levels (e.g., a body structure impairment is identified as a personal factor) (20). Nguyen et al. (25) suggested that full implementation and translation of the ICF in rehabilitation is impeded by current lack of clarity about the specific impact of personal factors on disability. Thus, there is a need to more precisely define personal factors and understand how their interaction with environmental factors creates the contextual conditions where function and disability develop. Without a comprehensive understanding of the entanglement of personal and environmental factors, the ICF risks a disregard for the life context of an individual, limits autonomy and subjectivity, and can be viewed as “unhumanized” (20, 24, 26, 27).

There is a clear knowledge gap related to how personal factors interact with environmental factors in shaping the contextual conditions that, according to the ICF, are critical for explaining the functioning and disability experienced by individuals with a given condition. This paper aims to provide a theoretical and philosophical foundation for rehabilitation scientists to address this important knowledge gap. In particular, we explore how a newly proposed philosophical model of disability from enactive cognitive science and ecological psychology, the *Ecological-Enactive Model of Disability* (9), can elucidate the role of personal factors in disability and functioning in rehabilitation. We first present the Ecological-Enactive Model of Disability and highlight how its conceptual framework can enhance understanding of how personal factors affect the disability experience. Our stance is supported by a review of evidence and phenomenological interviews from individuals with Friedreich's Ataxia (FA), emphasizing the Ecological-Enactive understanding of personal factors. We further consider the practical impact of this understanding on disability and rehabilitation research and pathways for the future of rehabilitation research focusing on representative design.

## The Ecological-Enactive Model of Disability

Toro, Kiverstein, and Rietveld (9) introduced the *Ecological-Enactive Model of Disability*, which can facilitate understanding of the interaction of personal and environmental factors that is critical for a complete, humanized description of the context in which disability develops. This model combines theoretical concepts from ecological psychology (28) and enactive cognitive science (29, 30) to explain disability in terms of a person's experience when they engage their bodies in meaningful activity. Like the ICF, the Ecological-Enactive Model locates these experiences not within the individual but in their intersection with the various contexts in which they are situated. Importantly, the Ecological-Enactive Model provides concepts and constructs that highlight the inextricable connection between personal and environmental factors that define the context shaping an individual's functioning and disability.

Proponents of the Ecological-Enactive Model suggest that the experience of disability arises when the skills that a person expresses are often and persistently insufficient to support their activities and social roles. Put differently, the experience of disability can be equated with frequent and pervasive experiences of “I-cannot.” According to the Ecological-Enactive Model, these experiences can only be understood if we give careful consideration to the relation between the action capabilities a person currently expresses and the social-institutional-physical environment in which they must behave. To be precise, the skills that a person has or lacks are a product of the extent to which they are able to leverage their action capabilities to discover and explore opportunities for activity available in their environment to fulfill their goals, wishes, and desires. Thus, the Ecological-Enactive Model invites us to understand the experience of disability as both situated (context-dependent) and embodied (shaped by the body and its capabilities).

We specifically draw on radical embodiment perspectives from both ecological and enactive approaches rather than the original concept of embodiment (31, 32). The conceptualization of embodiment in this perspective is critical for our current purposes because it conveys the important idea that what a given environment offers to a person cannot be defined objectively and independently of their lived experiences in their body. Rather, current action capabilities are co-constructed and co-defined by the relation between an individual's body, their goals, and their environment. For example, hills appear steeper when individuals are fatigued or wearing a heavy backpack (33) and may induce breathing difficulties for individuals with unilateral vocal fold paralysis (34). In short, the world is experienced differently, and it has different meanings, for individuals with differing bodies and bodily skills.

Proponents of the Ecological-Enactive Model borrowed the concept of *affordance* from ecological psychology to capture the entanglement of the individual-environment in defining the skill-based experiences that underlie disability. Affordances are possibilities for action that an environment offers to individuals given their capabilities (35). This concept is important because it describes environments in action-referential terms— that is, in relation to bodily skills. As a result, affordances can form the basis for explaining a person's experiences of “I-cannot.” In particular, activities are supported by available affordances that must be discovered (perceived) and actualized. A tall stair step, for instance, might be perceived as climb-able (i.e., affords climb-ability) to an individual with a particular body structure and capacity (e.g., the lower limb must be long enough, and the strength of lower-limb extensors needs to be sufficient to pull body mass upward). For an individual who does not meet these requirements, the stair step may not afford climb-ability, at least not in the typical way (36). Of course, when faced with such an “I-cannot” experience, a person can explore alternative ways to use their body or leverage environmental support to achieve the desired effect. For instance, a toddler might decide to crawl up the stair step while an adult with insufficient strength might use a cane.

Affordances “invite” behavior to different degrees depending on the goals and characteristics of the individual (i.e., personal factors) in that environment, which can help explain individual differences in and across disabilities that result in different motor solutions (37, 38). The concept of affordance allows one to appreciate that a person's experiences of disability cannot be reduced either to their bodily impairments nor social context alone. These experiences will necessarily depend on the extent to which they can use their capabilities to discover novel ways of exploring affordances, in order to have their needs and goals fulfilled. Importantly, the surrounding social-material-institutional world constrains the extent to which an individual experiencing disability can adapt. In sum, affordances inviting action will differ for each individual based on their specific physical capabilities and context.

Further, an individual with particular motor impairments may not be precluded from exploring specific affordances, but the motor patterns by which they actualize them may differ from those without motor impairments (39). This realization suggests that rather than evaluating behavioral patterns (movement patterns, speech patterns) in terms of how similar they are to “typical,” one should evaluate the extent to which behavioral patterns are adaptable to fulfill functional outcomes (39). For example, individuals modify their speech when speaking in a noisy environment, slowing their speech rate, increasing their vowel duration, and increasing acoustic energy in higher frequencies (40, 41). These articulatory and respiratory adjustments assist listeners in understanding speech in challenging listening situations, and the ability to adapt speech movements is a product of experience in listening and talking under different environmental conditions (42).

Adaptation is based not only on past experiences but also on the ability to implement novel solutions in changing contextual conditions and in new situations (9). Proponents of the Ecological-Enactive Model introduce the notion of bodily normativity on the basis of this adaptive capacity that individuals express to leverage environmental support in a variety of ways to preserve function. This contrasts with the reductive strategy of defining bodily normativity with respect to average characteristics of body structures and functions (9). Based on this understanding of bodily normativity, an individual with disability may experience “normal embodiment” if they demonstrate adaptability to a range of changing situations (perhaps through the exploration of seemingly “atypical” motor patterns) to achieve some environmental outcome. Pathological embodiment, on the other hand, would be experienced by individuals who are unable to adapt to change and, as a consequence, place themselves in more controlled situations bereft of change (9). Limiting adaptability and only experiencing familiar and controlled situations certainly shapes the disability experience.

The conceptualization of “atypical” behavior of individuals with disability as potentially adaptive is critical to the Ecological-Enactive Model because it avoids the temptation to pathologize the body for its structural and functional differences. Rather, it proposes that disability does not implicate pathological embodiment despite all the impairments in structure and function that might be objectively documented. In fact, individuals with disability often demonstrate “normal embodiment” in the sense that they are able to adapt their behavior to meaningfully engage with the environment using the affordances that work for them (9). For example, children with cerebral palsy (CP) demonstrate flexible patterns of grip control as upper-limb task demands change (43). Individuals with CP and stroke exhibit gait patterns that deviate from biomechanical “norms,” however, these patterns facilitate adaptability to changing contextual conditions (44) and make use of available action capabilities (45). In the context of speech, people with dysarthria may achieve better intelligibility and functional communication by slowing their speech rate “below normal” (46).

Normal embodiment does not mean that there is an absence of difficulty in performing daily tasks, but rather, it entails the ability (at the intersection of the individual and their context) to explore solutions when faced with such challenges. Because individuals with disability can experience normal embodiment, disability becomes a form of self-experience, constantly tackling the notions of “I-cannot,” and overcoming those feelings through the discovery of affordances that allow for the preservation and development of function and moments of “I-can” (9). This conceptualization of disability is not meant to minimize its challenges and the need to create accessible environments. Constantly overcoming moments of “I-cannot” through discovery of novel ways to move is energy- and time-consuming. Thus, shaping the physical and social environments to minimize the need of individuals with disability to adapt in order to achieve their functional goals is critical to promote their full participation.

## Personal factors in rehabilitation considering the Ecological-Enactive Model of Disability

The interaction of an individual's physical capabilities with their surrounding environment defines what affordances are available and can be explored to support the performance of meaningful activities. As argued above, physical impairments are certainly part of an individual's disability experience, but these impairments do not necessarily define *access* to particular affordances, and, therefore, cannot fully explain an individual's disability experience. Individuals can adapt their movements and leverage environmental support in a variety of ways to preserve function and skew pathological embodiment. The extent to which an individual can do this is shaped in important ways by personal factors. In particular, personal factors can alter the fit between an individual and their environment, defining the affordances available to support meaningful activities or social roles (39) and giving rise to either pathological or normal embodiment. Personal factors are also related to an individual's readiness to respond to the invitation of an affordance (i.e., “I-can”). Critically, affordances are not equitably distributed to all members of society.

Consider, for instance, an individual with a spinal cord injury (SCI) who went through a rehabilitation program where they learned to locomote using a wheelchair. Now imagine that this individual lives in a Brazilian favela. This individual will likely not be able to commute independently to and from work because the steep, uneven terrain leading to their home in the favela does not afford locomotion with a wheelchair. Now consider a second individual with the same SCI, who went through a similar rehabilitation program but lives in an affluent area. This second individual would not face the same limitations because they would have access to sidewalks and adapted cars that afford locomotion using a wheelchair. Pathological embodiment is thus avoided in the latter scenario because adaptation to the new bodily conditions is possible and effective. There is no question that the impact of environmental factors (e.g., accessibility) on disability is well-recognized. What the Ecological-Enactive Model invites us to notice is the fact that an individual's experience of their environment largely intersects with personal factors. Personal factors, like socioeconomic status, for instance, can define the kinds of environmental barriers or facilitators that a person is likely to encounter (in addition to access to resources and tools to overcome such barriers). Thus, personal factors are critical in the understanding of function and disability, and according to the Ecological-Enactive Model, their role should be considered in light of their impact on available affordances. We recognize the inherent simplicity of the above example, and it is important to note that other contextual factors can shape the disability experience. For instance, the individual living in the favela may have ample family support, whereas the individual in the affluent area may live alone. The goal of our example is to highlight the complexity of both environmental and personal factors in shaping an individual's disability experience and the affordances available.

In what follows, we present (a) evidence from physical therapy, occupational therapy, and speech-language pathology and (b) phenomenological interviews of individuals with FA that highlight the intersection of personal and environmental factors. We discuss how the entanglement of personal and environmental factors impacts functioning and disability.

### Evidence of entanglement of personal factors and environmental factors

In this section, we consider how various personal factors interact with environmental factors to define the context in which functioning and disability occur and develop. We specifically focus on how that interaction may skew motor and related rehabilitation outcomes. For example, women and girls have been theorized to “self-objectify” by adopting a third-person perspective of their bodies due to socialization and sexual objectification imposed by Western culture (47). Women and girls may transition from a subjective sense of self-as-agent to a sense of self-as-object (48). The sense of self-as-object pulls attentional focus to how the body looks or should be moving (i.e., internal focus of attention). An internal focus of attention has been shown to negatively impact motor performance and learning (49). Thus, self-objectification can alter skilled motor performance in women and girls through the disruption generated in attentional focus during activity (48).

In feminist philosophy, Young (50) famously argued that self-objectification accounts for inefficient body mechanics during the performance of multiple motor activities by women and girls, and differences cannot be attributed purely to physical and structural differences. One paradigmatic example is throwing (i.e., “throwing like a girl”): Girls do not use their whole bodies and typically restrict bodily movement to the arm while the rest of the body remains relatively immobile. In other motor behaviors like gait, women limit the amplitude of their movements compared to men (e.g., decreased stride in proportion to body) (50). Restricted bodily movement in this case is not pathological; it is due to the context in which individuals are embedded. Self-objectification may contribute to how women or girls with disability adapt their bodily movements and thus increase their likelihood to experience pathological embodiment.

There are also examples in speech-language pathology where particular behaviors are related to personal factors and not necessarily pathology. For example, some American English innovations, like glottal fry, continue to be stigmatized due to its (unconfirmed) association with young women (51). Glottal fry, often perceived as “creaky” voice, can indeed be a marker of functional or structural pathology and, in such a case, is likely to be persistent and out of the individual's control. For some speakers, however, it is used to signify syntactic information or serves as a sociolinguistic marker of group identity. There is no evidence that glottal fry as a sociolinguistic variant contributes to structural pathology, yet there are calls for women to abandon glottal fry (52). These prescriptivist views pathologize what is actually normal sociolinguistic variation (53).

The previous examples illustrate how personal factors (e.g., gender) interact with environmental factors (e.g., social norms, attitudes) to create possible detriments to motor performance (at the levels of body functions and activities and participation) by limiting the exploration of environmental affordances. The discussion of glottal fry also highlights how normal variation for individuals with particular personal factors can be artificially pathologized. In individuals with disability, these effects can multiply if increased focus is placed on a reductive, body-as-object perspective which emphasizes bodily impairments (54) and can thus create a state of pathological embodiment. Toro and Martiny (54) suggest that when focus is placed extensively on “properly performing” a task for individuals with CP, alternative movement strategies may not be explored.

Relatedly, stereotype threat—the concern arising when an individual from a stigmatized group feels at risk of confirming a negative stereotype (55)—has been shown to negatively impact motor performance. Many stereotypes that arise are related to personal factors. Gender-related stereotype threat, for instance, has been found to debilitate female performance on various athletic skills (56–60). Further, race-related stereotypes can lead to reduced motor performance in the stigmatized group (57, 61). Stereotype threat is thought to impede performance because individuals may increase cognitive performance monitoring which can subsequently disrupt task execution (62). Like self-objectification, stereotype threat enhances a body-as-object awareness, limiting the exploration of affordances necessary for the development of normal embodiment. Individuals with disability may additionally experience stereotype threat associated with their disability (63). The entanglement with other personal and environmental factors that generate stereotype threat can create the conditions necessary for a state of pathological embodiment for an individual with disability and feelings of “I-cannot.”

As a final example of the entanglement of personal and environmental factors, we explore interactions between therapists (i.e., clinicians and researchers) and recipients of care. Therapists are fundamental to the disability context: They shape how recipients of care view themselves (64), and personal factors may modulate the ability to interact and co-act. An objective, mechanistic focus on function and impairment during an interaction can result in successful task outcomes but can, at the same time, be ultimately dissatisfying and awkward experiences for an individual with disability (54). When therapists engage with mutual affordances and acknowledge the other's experiences, interactions are more satisfying than when a focus is placed on accomplishing a goal as efficiently as possible (54). Promoting the most affordances available for a recipient of care can become an important goal of the researcher/clinician. However, this may be less likely to happen if there is a large mismatch between identities such that mutual affordances are less likely to be discovered. In short, interactions are more satisfying when the researcher/clinician can facilitate “I-can” rather than forcing individuals with disability to tackle “I-cannot” through idealized action strategies.

### Lessons from Friedreich's Ataxia

In this section, we draw from phenomenological interviews conducted by the second author with individuals with Friedreich's Ataxia (FA), a progressive neuromuscular disorder that impairs motor coordination and balance for walking, speech, and hand function (65). Individuals interviewed were part of a larger study investigating the impact of FA on their daily life (66), and complete methodological details, including interview methods, procedures, coding, and data analysis can be found in that work (66). An overview of the methodology can be found in Table 1. In the current contribution, we focus on individuals who specifically discussed the impact of fatigue on their social interactions, and the data revealed meaningful examples that illustrate how environmental and personal factors interact with physical limitations to shape the disability experience. Phenomenological interviews are based on experience contextualization (67). Individuals are made aware of specific aspects of their experience, and they reflect on how they experience their bodies and their interactions with others and the environment (68). We use these illustrative findings to support understanding personal factors through the Ecological-Enactive Model of Disability.

**Table 1:** Overview of phenomenological interviews.

**Participant Information**	*n* = 13 (7F, 6M) Age at interview: 20-64 years Age at diagnosis: 13-51 years old Race: 13 Caucasian Ethnicity: 1 Hispanic; 2 Middle Eastern
**Interview Setting**	Interviews were conducted virtually *via* WebEx with participants and researcher located in a quiet room
**Guiding Interview Questions**	• Describe a typical day for you. • Describe your exercise routine (e.g. how many times/week do you exercise; what activities do you do?). • What is the most difficult part of your day? • How would you describe your fatigue to someone who doesn’t know FA? • What can you not do because of fatigue? • Describe a time when you did not do something because you were too tired. • What causes/brings on fatigue for you? • What helps to alleviate fatigue once it is there? • What do you do to prevent yourself from getting fatigued? • What would you consider to be an improvement in fatigue/energy level? • Describe how you feel when you think you’re fatigued. • Do you feel fatigue the same way all the time?
**Primary Coding Method**	Interviews were transcribed by a trained research assistant. The interviews were independently coded for themes by the interviewer (author CS) and the research assistant. The two coders discussed any disagreements in codes until a consensus was reached. Data collection continued until saturation of themes was achieved. Full coding details can be found in larger study (66).
**Data Extraction for Current Paper**	Quotations in which participants discussed their disability with respect to environmental and personal factors were identified by the second author (CS) from the larger study results (66). All authors reviewed and agreed upon the final selection and interpretation of quotations included in the current paper.

One individual interviewed remarked that having a disability is cognitively fatiguing due to the persistent need to focus on safety and planning for activity, such as a route to the bathroom. In other words, constantly overcoming moments of “I-cannot” is energy-consuming. She explained that, while her life is accessible because she has access to wheelchair ramps, the most difficult part about getting around with disability is

*“… just how taxing it is and how time-consuming and how much effort it takes to do it, like it should be really easy just to get up and get yourself a cup of water. But instead, you have to focus so much energy on that one simple task.”* (P3)

Another individual described the tools and equipment she has for mobility impairments, including a car lift, mobility stander, as well as nursing assistants and contractors to help with physical work around the house. Yet, even with these measures, the most difficult part of her day is “*fitting in everything I want to do…because I move so very slowly”* (P13). She further explained that she will wait to do activities that require more cognitive effort until her “best times” when she is not physically or mentally fatigued:

*“I have to manage my fatigue in that, you know, the things that I can do (which are limited), but the things that I can do, I have to, you know if I want to do something where I require a lot of thought processes. I know I can’t do that first thing in the morning until everything gets going …I am at my best probably late morning to midafternoon and then toward late afternoon, I start to slow down. So, I realize that I have to pretty much schedule those things that require me to be at my best during those hours, and the things that are easier for me to concentrate on or accomplish I do that in those times around my best time.”* (P13)

This account illustrates how factors in the environment (e.g., time of day) interact with physical limitations to influence a person's fatigue and their disability experience. It also shows individuals adapting movement to find optimal times for more moments of “I-can.”

Individuals with FA were conscious of how interlocutors may perceive them. One individual reflected that he worried about being taken seriously by vendors or customers at his job because of the way he walks and talks. He worried that others might think less of him or judge his character harshly because of the bodily impairments they might notice. Returning to the concept of stereotype threat, these concerns must be balanced with the constant demands on a person during their workday, presenting additional challenges to mental load or concentration. In trying to move “normally,” the individual reported seeking more order and control, limiting potential exploration of available affordances in ways that optimally work for him. These contextual conditions alter the affordances available and create more moments of pathological embodiment and “I-cannot”:

*“For me, because I am still independently mobile, there's a component where physically I want to make sure that where I’m at, I'm safe. I'm balanced. I'm in control of where I am and how my body is working. But then there is also the mental and the social side where I don't want to look too shaky or too different than everyone else. So, I try to have more control of my awkward moments, so I fit in a little bit…'* (P4)

Some individuals remarked that, although they found little benefit to their physical abilities by using a cane or a walker, they found value in using an assistive device in certain social settings (e.g., a wedding) to signify to other people that they needed more time or space to get around. “*When I’m out in public, I use a walker to walk. At home, I don't… I mean I probably could leave the walker at home, but I just feel more comfortable with it”* (P7). This illustrates how socialization interacts with the environment to shape the affordances available. Social influences can create conditions for individuals with disability that limit feelings of safety, and individuals are confronted with placing themselves in more controlled situations (*via*, for example, use of an assistive device). Managing disability is not simply about negotiating physical impairments and potentialities in order to functionally move through the world and achieve “I-can” experiences. It is doing so *in light* of others’ perceptions and attitudes that can either promote or encumber this achievement.

## Implications for rehabilitation and disability research

The summarized evidence suggests that the entanglement of personal and environmental factors can create states of pathological or normal embodiment for individuals with disability. The presence or absence of particular personal factors can change the fit between an individual with disability and their environment, and thus, change the affordances available to support activity and functional movement. Because of this, equality or equivalency in rehabilitation measures and outcomes among groups with differing personal factors should not be assumed. For example, a female with a motor disability may demonstrate certain patterns of motor behavior partly related to stereotype threat or self-objectification occurring when working with a male researcher. This individual may restrict movement during testing which may reveal a pattern of pathological embodiment and limited adaptation to changing contextual conditions that might be related to their gender rather than their medical condition. Conversely, males with the same disability may not restrict their movement when working with a male researcher and demonstrate normal embodiment. Research results may subsequently be limited if representation across multiple personal factors is not achieved. Particular adaptations observed in an experiment may be due to a condition or due to how the condition intersects with personal factors. In the previous example, if a study only includes female participants, one might conclude that individuals with a particular motor disability demonstrate pathological embodiment and the inability to adapt to contextual change. On the other hand, if the sample only includes male participants, a different narrative may emerge. It is important to note that differences across culture, race, socioeconomic status, and education may result in a similar skewing of findings. Unfortunately, there is currently inconsistent reporting (or absence of reporting) of personal factors in rehabilitation literature across disciplines (69–75).

We contend that there is a fundamental need for rehabilitation research to be representative across multiple personal factors in order to examine their impact on the reported findings. Minimally, researchers must systematically characterize research participants in terms of personal factors and consider the possibility that findings might differ for groups with different identities. Further, given that researcher-recipient of care interactions and observations can also be influenced by personal factors (and subsequently impact motor performance), research teams should recognize that these interactions can also impact study findings. This recommendation extends beyond studies in which personal factors are central to the research question (e.g., “What is the relationship between mobility outcomes after stroke and socioeconomic status?”). Cross-sectional motor control research, therapeutic programmatic evaluation, and clinical intervention development also require a thorough reporting of personal factors, as research results may suggest pathological or normal embodiment because of some combination of personal factors interacting with various ICF levels. In translating evidence to clinical practice, certain findings may not be replicated partly because research samples are not representative across personal factors.

Limited reporting of personal factors in rehabilitation research is partially reflective of the medical model continuing to serve as the pervasive foundation for many research and rehabilitation practices (76). Individuals are often included in a study based on a medical diagnosis and receive a one-size-fits-all intervention despite potential variations in body structure and functions, activity limitations, participation restrictions, and personal factors that should guide the selection of interventions in clinical practice. Translation of results at the individual level (i.e., external validity) can be a challenge when groups are collapsed into aggregate means (77, 78). The undesirable consequence is that studies may not be helpful to a clinician in determining an efficient and effective combination of practices for the majority of their clients. Clinicians may deliberately dismiss findings because their clients are different from those included in the studies (79). Likewise, a clinician might apply an evidence-based intervention because it is supported by aggregate data, but it might not be appropriate for that individual considering differences in personal factors.

Fundamentally, rehabilitation services are often developed by and for majority groups (in terms of personal factors), and they may subsequently fail to meet the needs of minority groups (72). The potential adverse outcome is the generation of “standard” assessments and interventions that are not appropriate for specific individuals given their multiple personal factors and how those personal factors interact with other ICF levels. Ensuring that samples are representative across multiple personal factors can facilitate external validity of research results as well as mark a critical step in emphasizing the role of personal factors in altering the fit between an individual and their environment in rehabilitation.

### Barriers to representative design

A multitude of research participation barriers for individuals with a variety of personal factors have been identified, particularly for those from historically underrepresented groups. Researchers often lack an understanding of personal factor differences, resulting in ineffective communication for recruitment, enrollment, and retention (80, 81). Researchers may fail to facilitate the informed consent process to ensure that participants truly make informed decisions about their participation. Mistrust and fear from a dark history of systemic abuse and exploitative medical research targeting racial and ethnic minorities in the United States continue to act as substantial barriers to research participation (80, 82–84). Concerns related to childcare and transportation can also interfere with successful recruitment and retention, particularly for women and individuals of low socioeconomic status (80, 81, 83, 85, 86).

Women and other gender minorities may be inadvertently excluded from studies in which eligibility criteria are focused on a male pattern of presentation for a particular diagnosis (86, 87). For example, age-based exclusion criteria disproportionately affect women in stroke clinical trials (88). Individuals in the transgender community commonly cite researcher mistrust and lack of awareness of research opportunities as barriers to participation in health research. Transgender people question researcher motives, as they often feel that their community does not benefit from the research conducted. Some report negative prior experiences of being misgendered. Importantly, transgender people express emotional concerns about being “outed” (81). Owen-Smith et al. (81) also identified that while individuals often appreciate a financial incentive, this can act as a barrier to research participation. If individuals are required to obtain public transportation, this cost is removed from, and thus reduces, their research compensation. Further, a gift card (rather than a cash incentive) restricts where participants can spend the incentive, which can be limiting for individuals of low socioeconomic status.

Cultural minorities may experience language barriers, limited social support systems (particularly for immigrants and refugees), and difficulty navigating unfamiliar health systems (72). Language barriers can impact the development of researcher relationships with participants, and it may take longer to establish rapport (72). For immigrant Latinos in the United States, there is additional concern over immigration status and fear of deportation (80, 89). Parents from some cultural backgrounds may seek to conceal the disability of a child, as their culture indicates that disability is a source of shame (90). On the other hand, some cultures value disability as adding to the diversity of society (91). Individuals from each of these cultural backgrounds may be reluctant to seek services or to participate in research because of their culture's views on disability.

The barriers addressed in this section are coupled with barriers for recruiting individuals with disability—social devaluation, inaccessible recruitment materials, a digital divide, and power differentials (92)—creating a multiplicative effect and poor representation of individuals with a diversity of personal factors in the rehabilitation literature.

### Paths forward: recommendations

#### Recruitment, enrollment, & retention

Increasing diversity among personal factors in rehabilitation research is largely contingent on effective recruitment strategies. Researcher actions involving authentic and long-term community engagement and community-based groups can help the researcher build rapport and trust with potential participants and facilitate enrollment (80, 93–96). Recognizing that individuals with disability have less access to technology (92), creating recruitment materials that are publicly available (97, 98) and available in multiple languages at the appropriate readability level can help in recruitment (97).

Consent is a critical step in rebuilding trust between researchers and the community. The researcher may need to read the informed consent aloud to facilitate active questioning (98). In multiple-session studies, the informed consent process should be an ongoing discussion, which can also aid in retention (80, 85, 99). Participatory action research (PAR) and community-based participatory research (CBPR) approaches could also be considered to aid in the recruitment, inclusion, and retention of diverse communities with disability (100, 101). PAR, CBPR, and related approaches allow community members to act as researchers, assisting with collecting, analyzing, and interpreting data, and learning the technical tools of research to further facilitate collaboration (101). PAR can also give voice to whether implementation of research and healthcare practices are meeting the needs of individuals with disability (102).

Increasing the diversity of the research team across multiple personal factors can facilitate more representative research. In certain cultures, a clinician/researcher of the same gender as the recipient of care may facilitate comfort at the site (72). Transgender individuals have reported that they would be more likely to participate in research studies if the researcher involved in recruitment or data collection was transgender (81), or if the staff has undergone specific training on transgender issues and vocabulary to actively prevent undesirable research experiences. Similarly, women may be more likely to participate in clinical research if a female researcher leads the project (103).

Although critically important, language is only one aspect to consider in facilitating comfort at a research or clinical site. For example, a Spanish-speaking person functioning as an interpreter for a Spanish-speaking participant may be from a different socioeconomic group, speak a different dialect, or not be able to summarize the research in participant-focused terminology. Horton and Munoz (104) recommend that, in addition to including linguistically-diverse research staff or interpreters to assist in consent or data collection, clinicians/researchers use cultural adaptation models to tailor evidence-based treatments to specific contextual and individual aspects of cultural identity in participants. For example, the Ecological Validity Framework seeks to identify the way cultural properties of a treatment correspond to a person's cultural linguistic experience, as well as the researcher's assumptions (105). If threats to ecological validity are discovered, the clinician/researcher can adapt the intervention to achieve better congruence with the participant's cultural identity and environment.

Researchers should consider working with an individual with disability and including this individual in the design and implementation of the study (106). Including a researcher with disability may improve the research experience for individuals with disability and challenge ableist norms. We recommend comprehensive involvement of the population being investigated for any disability study. Researchers should seek to facilitate “I-can” for individuals with disability, rather than only the individual with disability having to tackle “I-cannot.” For example, researchers can make sure to highlight accessible routes to their lab rather than forcing the participant to try multiple avenues that lead to feelings of “I-cannot.”

Providing incentives and options for a financial incentive (e.g., cash, food, transportation, donation) can facilitate recruitment and demonstrates researcher understanding of complex social issues (e.g., food insecurity) and is generally appreciated by individuals being recruited (81). Flexible options for scheduling, including weekends and weekday evenings, similarly demonstrates a concerted effort by the researcher to work with potential participants to facilitate enrollment while remaining sensitive to participant employment demands (81).

#### Study design

Comparative effectiveness research (CER) is an emerging research method that can better integrate personal factors into scientific investigations (79, 107). The approach analyzes the relationship between interventions and individual outcomes, recognizing that sample heterogeneity and intervention combinations must be considered for meaningful intervention comparisons (107). One specific CER strategy, practice-based evidence for clinical practice improvement (PBE-CPI), is an observational study design that captures comprehensive information related to individual characteristics (e.g., body mass, functional status), care processes (e.g., medication management, nutritional support), and outcomes (e.g., falls, change scores) while controlling for individual differences (79, 107). The approach explicitly acknowledges that regular clinical practice involves individuals receiving multiple interventions concurrently. Small, nonsignificant effects of an intervention may become significant when combined with other interventions. Further, and relevant to the argument of the current paper, the effectiveness of intervention combinations may differ based on the individual and the individual's personal factors. With PBE-CPI, hypotheses are general, multiple interventions are considered to capture treatment differences that exist in clinical practice, participant recruitment criteria are minimal, individual differences are controlled through statistics (as opposed to randomization), emphasis is placed on transparency, and the transdisciplinary team is prioritized (79). Rather than suppressing personal factors by attempting to create a homogenous sample that may lack external validity, CER embraces the diversity of personal factors.

When preparing any study for publication, authors should report the personal factors of the sample (what can be collected), aiming to achieve data collection within the distribution representative of the specific disability under consideration. If researchers are unable to achieve this distribution, they should report this as a limitation to the study. Reviewers and editors are encouraged to hold authors accountable to these (very minimal) standards as an initial first step toward better recognizing the role of personal factors in rehabilitation study results.

## Discussion

This paper described how the Ecological-Enactive Model of Disability can be used to make sense of the role of personal factors and the interaction of personal and environmental factors in shaping the motor performance of individuals with disabilities with implications for rehabilitation research. The Ecological-Enactive Model avoids pathologizing the body by considering disability as a form of self-experience in context, rather than a result of structural, functional, or social differences alone. Individuals with disability can exhibit normal embodiment in the sense that they are able to change and adapt motor patterns by using the affordances that work for them. Pathological embodiment arises when there is a resistance to or impossibility of change (9). Personal factors interact with environmental factors to define the affordances available and invite opportunities for action, which may create or preclude the development of pathological embodiment and moments of “I-can” or “I-cannot.” We proposed that this critical influence of personal factors requires a systematic characterization of research participants in terms of personal factors and consider that research findings might differ for groups with different identities.

Many former and ongoing debates about the ICF are centralized on the framework pathologizing the body and on its minimization of personal factors. Many disability scholars believe that the ICF places disability as a problem located within the individual which subsequently lends itself to rehabilitation professionals “fixing” the individual, rather than adjusting the environment to meet their needs (19, 91, 108). Specific to personal factors, there is concern that increased specification and categorization of these factors may result in blaming individuals with disability for their impairments and limitations (23). The Ecological-Enactive Model allows for the various ICF levels to be understood with an emphasis on the disability experience, as disability does not necessarily require pathological embodiment (9). Further, the model intrinsically couples an individual to their environment, and personal factors can alter the individual-environment fit.

The Ecological-Enactive Model also provides a fruitful basis for understanding the fluidity of the two components comprising context in the ICF: environmental and personal factors. Environmental factors are viewed as “external” to an individual, and personal factors are “internal.” However, many “internal” personal factors are manifested in an external world and shape subsequent interactions in the world. Gender can be conceived as an internal personal factor, for example, but the external expression of gender can impact how one is perceived in the external world and how those in the world respond to the person. Further, that external expression, combined with personal coping strategies and attitudes, can impact participation and body structures and functions for an individual with disability. Fundamentally, a web of determinants shapes the disability experience. In particular, it shapes whether an individual demonstrates pathological or normal embodiment, which may vary day-to-day due to the shifting demands of one's environment. Personal factors are often evaluated independently and in isolation from other ICF levels and environmental factors on outcomes of interest (e.g., impact of socioeconomic status on health outcomes). The Ecological-Enactive Model helps to make sense of the interactions inherent to the ICF rather than assessing each level individually. Personal factors intersect^1^ and are entangled with every ICF level. Recognizing this entanglement and generating representative samples can strengthen research practices and improve knowledge translation in multiple areas of rehabilitation.

While writing this paper, one of the authors of the current contribution with FA raised an important question, and we challenge readers to also ask the same question: “*How do people without disability use this information to better relate to those with disabilities?”* Individuals conducting rehabilitation research should build trust with participants with a diversity of personal factors, but efforts should go beyond trust. Researchers must facilitate “I-can” for individuals with disability, rather than giving sole responsibility to individuals with disability to struggle with notions of “I-cannot.” For example, if a research environment is not accessible, it cannot be a responsibility given only to the individual with disability to confront this inaccessible environment (“I-cannot”) and find the affordances that work for them on their own. The researcher/clinician plays a joint role in the effort and must be aware of the lack of accessibility to proactively provide more moments of “I-can.” Doing so may catalyze the involvement of individuals with disability in rehabilitation research while concurrently establishing a trusting relationship.

We wish to emphasize that there is still much work to be done in examining the interaction of personal factors in rehabilitation. Many of the examples identified in this paper examine rigid personal factor categorizations, which themselves may be inherently more nuanced. For example, demographic questionnaires may restrict gender identity to a male/female binary, ignoring the unique experiences of those elsewhere on the gender spectrum. The goal of this paper was to highlight the importance of personal factors, contextualize personal factors in the environment, and provide examples and recommendations for research practices. As such, some personal factors mentioned may require finer gradation in implementation, and there are other factors not mentioned here worth examination and inclusion.

## Conclusion

Disability is an experience that emerges from the dynamic interaction between an individual with a health condition and the context in which they are embedded. Personal factors are central to this context. The Ecological-Enactive Model of Disability provides a framework for better understanding the role of personal factors on function and disability by deeming disability a form of self-experience, considering the affordances available given an individual's personal factors. Strengthening future rehabilitation research and interventions critically depends on careful attention to personal factors.

## Data Availability

The original contributions presented in the study are included in the article, further inquiries can be directed to the corresponding author/s.

## Footnotes

^1^In this paper, we emphasize the interactions among the various ICF levels, focusing specifically on interactions between environmental and personal factors. While tacit in our account, it is important to more explicitly note that there are also intersections within ICF levels that can create a multiplicative effect. Intersectional Theory (109) posits that overlapping social identities (e.g., race, gender identity, socioeconomic status) relate to structures of oppression and discrimination. While beyond the scope of this paper to fully discuss this theory, we acknowledge the relevance of Intersectional Theory to the topics presented and encourage interested readers to further explore this theory and its relationship to disability experiences.
